# Establishing an appropriate *Z* score regression equation for Chinese pediatric coronary artery echocardiography: a multicenter prospective cohort study

**DOI:** 10.1186/s12887-021-02877-9

**Published:** 2021-09-30

**Authors:** Shu-min Fan, Bei Xia, Wei-xiang Liu, Wei Yu, Zhi-xia Wu, Shu-Bao Chen, Qing-Hua Liu, Wen-juan Chen, Shan-liang Zhu, Mei Jin, Jie-Qing Min, Yang Xu

**Affiliations:** 1 Ultrasound Department, Shenzhen Children’s Hospital Affiliated to China Medical University, 7019 Yitianroad, Shenzhen, Guangdong 518038 People’s Republic of China; 2 Shenzhen University, Shenzhen, China; 3 Shanghai Children’s Medical Center, Medical College of Shanghai Jiaotong University Shanghai, Shanghai, China; 4 Qilu Children’s Hospital of Shandong University Jinan, Jinan, Shandong China; 5 Children’s Hospital of Hunan Province Changsha, Changsha, Hunan China; 6 Nanjing Children’s Hospital, Nanjing, Jiangsu China; 7 Chengdu Women’s and Children’s Center Hospital Chengdu, Chengdu, Sichuan China; 8 Children’s Hospital Affiliated to Kunming Medical University Kunming, Kunming, Yunnan China; 9 The First Affiliated Hospital of Jilin University Changchun, Changchun, Jilin China

**Keywords:** Coronary artery disease, Coronary artery diameter, Echocardiography, Reference values, Z scores, Chinese ethnicity, Pediatrics

## Abstract

**Background:**

*Z* score utility is emphasized in classifying coronary artery lesions in Kawasaki disease patients. The present study is the largest such multicenter Chinese pediatric study about coronary artery diameter reference values and *Z* score regression equation to date. It is useful in Chinese pediatric echocardiography.

**Methods:**

A multicenter cohort was assembled, which consisted of 852 healthy children between 1 month and 17 years of age, ten children were excluded because their ultrasound images were not clear, or lost in following up. Diameters of the right coronary artery, left coronary artery, and left anterior descending coronary artery were assessed using echocardiography. Data were body surface area (BSA)-corrected using BSA calculated via either the Stevenson BSA formula or the Haycock BSA formula. Coronary artery diameter reference values and *Z* score regression equations were established for use in the Chinese pediatric population.

**Results:**

No difference was observed between coronary artery diameter data corrected using BSAste or BSAhay. Of the five assessed regression models, the exponential model exhibited the best fit and was therefore selected as the basis for derivation of the SZ method. When comparing *Z* scores, those produced by the SZ method conformed to the standard normal distribution, while those produced by the D method did not. In addition, there was a statistically significant difference between *Z* scores produced by the SZ and D methods (*P* < 0.05).

**Conclusions:**

Coronary artery diameter reference values for echocardiography were successfully established for use in the Chinese pediatric population, and a *Z* score regression equation more suitable for clinical use in this population was successfully developed.

## Background

Echocardiography is commonly used to evaluate pediatric patients for coronary artery disease (CAD). Kawasaki disease is the major cause of childhood-acquired CAD. Accurate pediatric reference values for echocardiographic monitoring of changes in coronary artery diameter contribute to establishing diagnosis and prognosis. The most recent American Heart Association guidelines recommend body surface area (BSA)-corrected coronary artery diameter as the gold standard, and emphasize *Z* score utility in evaluating coronary artery injury risk and in classifying coronary artery lesions in Kawasaki disease patients. The American Society of Echocardiography also recommends the use of *Z* scores in pediatric cardiology [1]. These scores indicate the distribution of measurements about the mean within a healthy population, and facilitate comparison of datasets exhibiting differential means and distributions (such as datasets deriving from children of different ages and sizes).

Coronary artery *Z* score regression equations have been derived for multiple healthy pediatric populations, including those of the United States [2], Canada [3, 4], Japan [5], Singapore [6], Turkey [7], Korea [8], and China [9]. With the advent of reference value predictive models, many manufacturers have incorporated such models into echocardiography equipment-integrated software in order to allow rapid computation of coronary artery measurement *Z* scores. However, whether models developed in North America and/or using different BSA formulae are suitable for use in the Chinese pediatric population remains unclear.

The present study therefore aimed to: (1) establish coronary artery diameter reference values in a healthy Chinese pediatric population, and (2) compare suitability for use in this population of a Z score regression equation derived from these data with that of one derived from data of mostly-Caucasian pediatric population [4].

## Methods

### Materials

A multicenter healthy Chinese pediatric cohort was assembled. Pediatric cardiologists and ultrasound experts from six provinces in Southern and Northern China participated in this study. All researchers reached a consensus and unified the inclusion and exclusion criteria. The cohort consisted of 862 participants between July 1, 2016, and December 30, 2019.The children within this cohort were followed up for 6 months. Ten children were excluded because their ultrasound images were not clear, or lost in following up. There were 546 males and 296 females. Height (H), weight (W) and systolic / diastolic blood pressure (SBP / DBP) were recorded. At each center, echocardiographic data of each child were evaluated.

#### Inclusion criteria

1. Age from 1 month to 18 years old.
2. No cardiovascular history.
3. Blood pressure was normal [10].
4. Physical examination showed no abnormality of cardiovascular system.
5. Heart structure were normal by clinical examination and echocardiography.
6. Echocardiography examination for the following reasons: asymptomatic heart murmur, chest pain, suspicious abnormal chest X-ray or ECG performance, family history of congenital heart disease.

#### Exclusion criteria

1. Congenital heart disease (The presence of a patent foramen ovale in infants was considered normal).
2. History of KD or surgical catheterization for heart disease.
3. Myocardial disease.
4. Chromosomal abnormalities (i.e. trisomy 21, Turner syndrome, noonan syndrome, Marfan syndrome, Williams syndrome, 22q11.2 deletion, etc.).
5. Major potential diseases (such as malignant tumor, leukemia, metabolic or endocrine diseases, neuromuscular diseases, chronic renal failure, etc.).
6. Professional athletes.

This study was approved by the ethics committee of Shenzhen children’s hospital. Signed the informed consent by children’s parents were obtained.

### Equipment and methods

The age, weight, and height of each patient were recorded at the time of the echocardiography evaluation. Data were body surface area (BSA)-corrected using BSA calculated via either BSA Stevenson formula (BSAste) [11] or the BSA Haycock formula (BSAhay) [12].

$$ \mathrm{BSASte}\left[{\mathrm{m}}^2\right]=0.0061\times \mathrm{H}\left[\mathrm{cm}\right]+0.0128\times \mathrm{W}\left[\mathrm{kg}\right]-0.1529 $$

$$ \mathrm{BSAHay}\left[{\mathrm{m}}^2\right]=0.024265\times \mathrm{H}{\left[\mathrm{cm}\right]}^{0.3964}\times \mathrm{W}{\left[\mathrm{kg}\right]}^{0.5378} $$

**Fig. 1:**
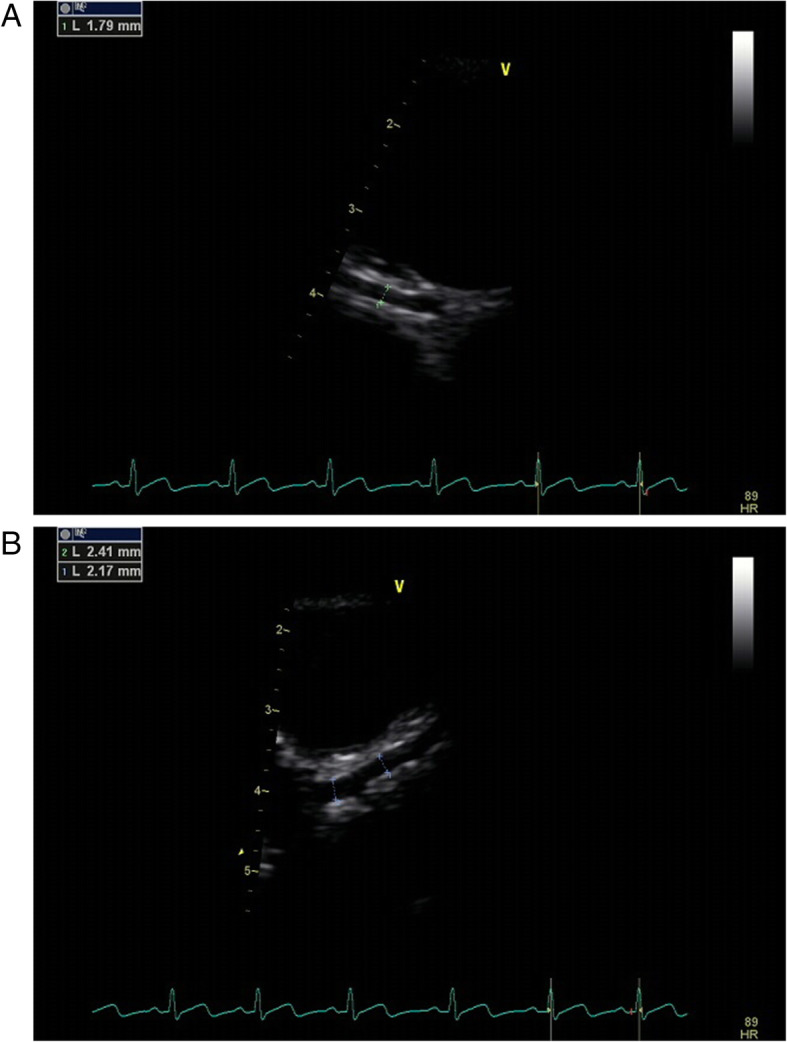
Short-axis echocardiographic view used for coronary artery diameter measurements. **A** RCA, right coronary artery; **B** LCA, left coronary artery; LAD, left anterior descending coronary artery

The studies were performed using GE Vivid (GE Healthcare, Milwaukee, WI) or Philips iE33 echocardiograph (Philips Medical Systems, Bothell, WA) echocardiography system. All Echocardiographic data were digitally recorded, allowing offline measurements. The frequency of the probe used should be as high as possible, the frame frequency > 60fps. Diameters of the right coronary artery (RCA), left coronary artery (LCA), and left anterior descending (LAD) coronary artery were assessed using echocardiography [13], which were defined as raw values (Fig. 1).

### Statistics analysis

General data: SPSS 11.0 software (SPSS, Inc., Chicago, IL, UnitedStates) was used for statistical analysis. Continuous variables (age, height, weight, blood pressure, heart rate, BSA) were expressed as mean (SD) and / or medians.

The Anderson darling test is used to determine whether the data conform to the normal distribution. The student’s t test was used to compare the difference between two groups of data which conformed to a standard normal distribution; Wilcoxon signed-rank test was used to compare the difference between two groups of data which did not. *P* values< 0.05 were considered statistically significant.

#### Regression analysis

Five regression models (linear, logarithmic, power, exponential, and squareroot) were assessed for goodness of fit using R language,based on adjustedR^2^, residual standard error (RSE), and Akaike information criterion (AIC) values. The best-performing model (maximum adjusted R^2^and minimumRSEandAIC) was carried forward to derive a novelreference regression equation, termed the ShenZhen (SZ) method.

#### Comparison of two regression equations

Both the SZmethod and the North AmericanDallaire(D)method [4] were applied to the same set of raw data, and resultant*Z* scores were compared.

## Results

### Cohort demographic data

Overall, a pediatric cohort of 842 healthy subjects was assembled, comprising 67% males and 33% females, and spanning an age range of 1 month to 17 years (Table 1).

**Table 1:** Age demographic information of healthy Participants

Age group	NO.	
	Female	Male
1mo - < 3mo	9	12
3mo- < 1y	40	78
1- < 2y	44	80
2- < 3y	30	57
3- < 4y	34	45
4- < 5y	23	40
5- < 6y	20	37
6- < 7y	15	32
7- < 8y	20	36
8- < 9y	12	26
9- < 10y	8	20
10- < 11y	11	20
11- < 12y	8	10
12- < 13y	0	3
-14y	2	3
-15y	2	1
-16y	3	1
-17y	0	0

*m* Month, *y* Year.

Echocardiography-determined coronary artery diameters corrected using BSAs calculated via two different formulae.

Both BSAste- and BSAhay-corrected coronary artery diameters were normally distributed (*P* > 0.05) and did not differ significantly from each other (P > 0.05) (Table 2).

**Table 2:** Comparison of coronary artery diameters corresponding to BSAste and BSAhay

BSA(m^2^)	BSAste			BSAhay		
	Mean(SD)			Mean(SD)		
	LCA(mm)	LAD(mm)	RCA(mm)	LCA (mm)	LAD(mm)	RCA(mm)
0.2- < 0.3	1.872(0.234)	1.523(0.199)	1.477(0.155)	1.765(0.257)	1.540(0.193)	1.467(0.179)
0.3- < 0.4	2.053(0.226)	1.702(0.205)	1.720(0.225)	1.993(0.221)	1.641(0.197)	1.668(0.235)
0.4- < 0.5	2.137(0.212)	1.786(0.210)	1.817(0.257)	2.151(0.219)	1.777(0.210)	1.792(0.231)
0.5- < 0.6	2.249(0.214)	1.858(0.206)	1.898(0.210)	2.233(0.219)	1.850(0.200)	1.888(0.224)
0.6- < 0.7	2.398(0.235)	1.978(0.215)	2.006(0.254)	2.391(0.227)	1.973(0.219)	1.988(0.251)
0.7- < 0.8	2.453(0.280)	2.024(0.233)	2.083(0.274)	2.462(0.305)	2.051(0.248)	2.117(0.292)
0.8- < 0.9	2.576(0.285)	2.134(0.239)	2.136(0.269)	2.569(0.286)	2.106(0.228)	2.101(0.248)
0.9- < 1.0	2.711(0.306)	2.189(0.226)	2.172(0.220)	2.704(0.299)	2.198(0.214)	2.206(2.538)
1.0- < 1.1	2.711(0.273)	2.204(0.223)	2.197(0.236)	2.754(0.285)	2.223(0.268)	2.193(0.274)
1.1- < 1.2	2.866(0.315)	2.373(0.241)	2.338(0.292)	2.843(0.342)	2.311(0.249)	2.311(0.253)
1.2- < 1.3	3.051(0.325)	2.430(0.175)	2.431(0.318)	2.955(0.282)	2.444(0.167)	2.446(0.279)
1.3- < 1.4	2.991(0.394)	2.464(0.214)	2.500(0.236)	3.042(0.383)	2.448(0.231)	2.462(0.301)

^a^ There were no significant differences between participants in the BSAste group and BSAhay group (*P* > 0.05)

### Regression model performance comparison

The R project for statistical computing was used to compare five regression models, including linear, logarithmic, power, exponential, and square root. Of these, the exponential model exhibited the best fit and was therefore selected as the basis for derivation of the SZ method. Exponential model performance based on BSAste-corrected values was as follows: lnyLCA = 1.0042 + 0.3494lnx (R^2^ = 0.678, RSE = 0.114, AIC = − 932.57), lnyLAD = 0.8013 + 0.3448lnx (R^2^ = 0.641, RSE = 0.120, AIC = − 849.8), and lnyRCA = 0.8107 + 0.3098lnx (R^2^ = 0.4806, RSE = 0.150, AIC = − 604) (Table 3). Exponential model performance based on BSAhay-corrected values was as follows: lnyLCA = 1.0084 + 0.3740lnx (R^2^ = 0.682,RSE = 0.115, AIC = − 938.7), lnyLAD = 0.8063 + 0.3697lnx (R^2^ = 0.650, RSE = 0.119, AIC = − 858.8), and lnyRCA = 0.8040 + 0.3291lnx (R^2^ = 0.4815, RSE = 0.150, AIC = − 598.2) (Table 3). Because the exponential model based on BSAhay-corrected values yielded the maximum adjusted R2 and minimum RSE and AIC, it was selected to construct a novel regression equation, herein referred to as the SZ method.

**Table 3:** Reference regression equation of coronary artery Echocardiography based on the BSAste and BSAhay formulas

BSA method	Coronary artery	regression equation	*R*^*2*^	*RSE*	*AIC*
BSAste	LCA	lny = 1.0042 + 0.3494ln x	0.6779	0.114	−932.57
	LAD	lny = 0.8013 + 0.3448ln x	0.6414	0.120	−849.8
	RCA	lny = 0.8107 + 0.3098lnx	0.4806	0.150	−604
BSAhay	LCA	lny = 1.0084 + 0.3740lnx	0.6823	0.115	−938.7
	LAD	lny = 0.8063 + 0.3697lnx	0.6503	0.119	−858.8
	RCA	lny = 0.8040 + 0.3291lnx	0.4815	0.150	−598.2

### Relationship of coronary artery diameter to BSAhay, and Z score distribution

**Fig. 2:**
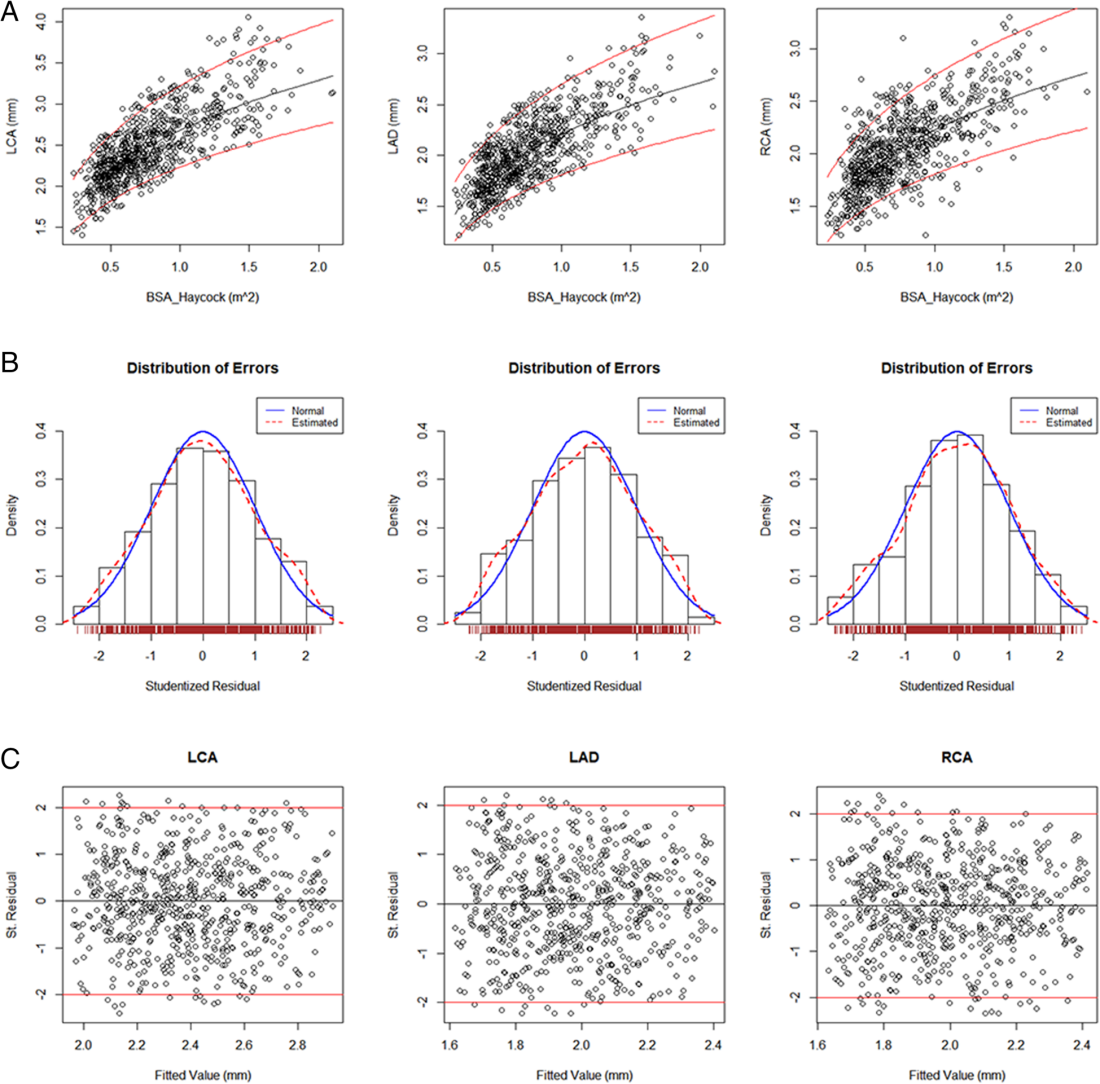
A1-A3: Trend charts and predicted mean curves for LCA, LAD, and RCA. The black line is the predicted value, and the red line is the prediction interval with 95% confidence. B1 ~ B3: studentized residual diagrams for LCA, LAD, and RCA. The black line is the normal curve, and the red line is the estimated curve. C1 ~ C3: Z score scatter plots for LCA, LAD, and RCA, which represent the fitted values of the regression models, along with upper and lower bounds, i.e. 2 and − 2 z-scores, respectively

Coronary artery diameters increased non-linearly with BSAhay (Fig. 2A). Values were normally distributed (Fig. 2B), and the normal distribution was standard (*P* > 0.05) (Fig. 2C).

### Comparison of SZ versus D method Z scores

When comparing *Z* scores resulting from the SZ versus D methods, SZ-derived *Z* scores conformed to a standard normal distribution, while D-derived Z scores (within certain BSAhay ranges) did not, and the difference between *Z* scores resulting from the two methods was significant (*P* < 0.05) (Table 4). Based on raw LCA diameter data, SZ method Z scores were larger than those of the D method when BSA ≤ 1.0 m^2^, but smaller than those of the D method when BSA > 1.0 m^2^ (*P* < 0.05). Similarly, based on raw LAD artery diameter data, SZ method *Z* scores were larger than those of the D method when BSA ≤ 1.3 m^2^, but smaller than those of the D method when BSA > 1.3 m^2^ (*P* < 0.05). Finally, based on raw RCA artery diameter data, SZ method *Z* scores were larger than those of the D method when BSA ≤ 0.7 m^2^, but smaller than those of the D method when BSA > 0.7 m2 (P < 0.05).

**Table 4:** Based on the same set of raw data, two groups result of Z scores calculated by SZ method and D method respectively

BSA (m^2^)	LCA -*Z* score		LAD -*Z* score		pRCA -*Z* score	
	SZ method	D method	SZ method	D method	SZ method	D method
0.2–0.3	0.104 ± 0.465	1.354 ± 0.921*	0.295 ± 0.395	1.761 ± 0.731*	−0.040 ± 0.256	0.979 ± 0.490*
~ 0.4	0.135 ± 0.329	1.178 ± 0.665*	0.140 ± 0.341	1.316 ± 0.616*	0.0869 ± 0.376	0.892 ± 0.751*^▲^
~ 0.5	0.133 ± 0.310	1.014 ± 0.655*	0.147 ± 0.345	1.207 ± 0.653*^▲^	0.084 ± 0.344	0.681 ± 0.681*^▲^
~ 0.6	0.033 ± 0.286	0.594 ± 0.806*^▲^	0.060 ± 0.314	0.579 ± 1.415*^▲^	0.049 ± 0.305	0.336 ± 0.758*^▲^
~ 0.7	0.052 ± 0.268	0.537 ± 0.576*^▲^	0.067 ± 0.311	0.804 ± 0.601*^▲^	0.037 ± 0.315	0.158 ± 0.621*^▲^
~ 0.8	−0.027 ± 0.355	0.261 ± 0.748*	0.024 ± 0.342	0.621 ± 0.651*	0.075 ± 0.357	0.071 ± 0.717
~ 0.9	−0.026 ± 0.328	0.142 ± 0.703*	−0.018 ± 0.309	0.440 ± 0.589*	−0.036 ± 0.319	− 0.299 ± 0.607*
~ 1.0	0.019 ± 0.334	0.038 ± 0.682	−0.013 ± 0.306	0.315 ± 0.560*	−0.042 ± 0.325	− 0.533 ± 0.606*
~ 1.1	− 0.065 ± 0.298	− 0.167 ± 0.646	− 0.103 ± 0.343	0.097 ± 0.649*	− 0.116 ± 0.325	−0.729 ± 0.577*
~ 1.2	−0.073 ± 0.362	− 0.26 ± 0.775*	− 0.081 ± 0.057	0.057 ± 0.619*	− 0.103 ± 0.361	−0.812 ± 0.631*
~ 1.3	0.041 ± 0.267	−0.301 ± 0.616*^▲^	0.002 ± 0.18	0.122 ± 0.365*	0.023 ± 0.289	−0.6943 ± 0.546*
~ 1.4	0.068,0.384	−0.426 ± 0.857*	−0.067 ± 0.251	− 0.082 ± 0.490	0.018,0.298	− 0.882 ± 0.560*

^▲^ according to Anderson darling test, *P* < 0.05 does not conform to normal distribution;

* *P* values for differences between the two groups of *Z* scores results calculated by SZ method and D method were less than0 .05

### D method Z scores

**Fig. 3:**
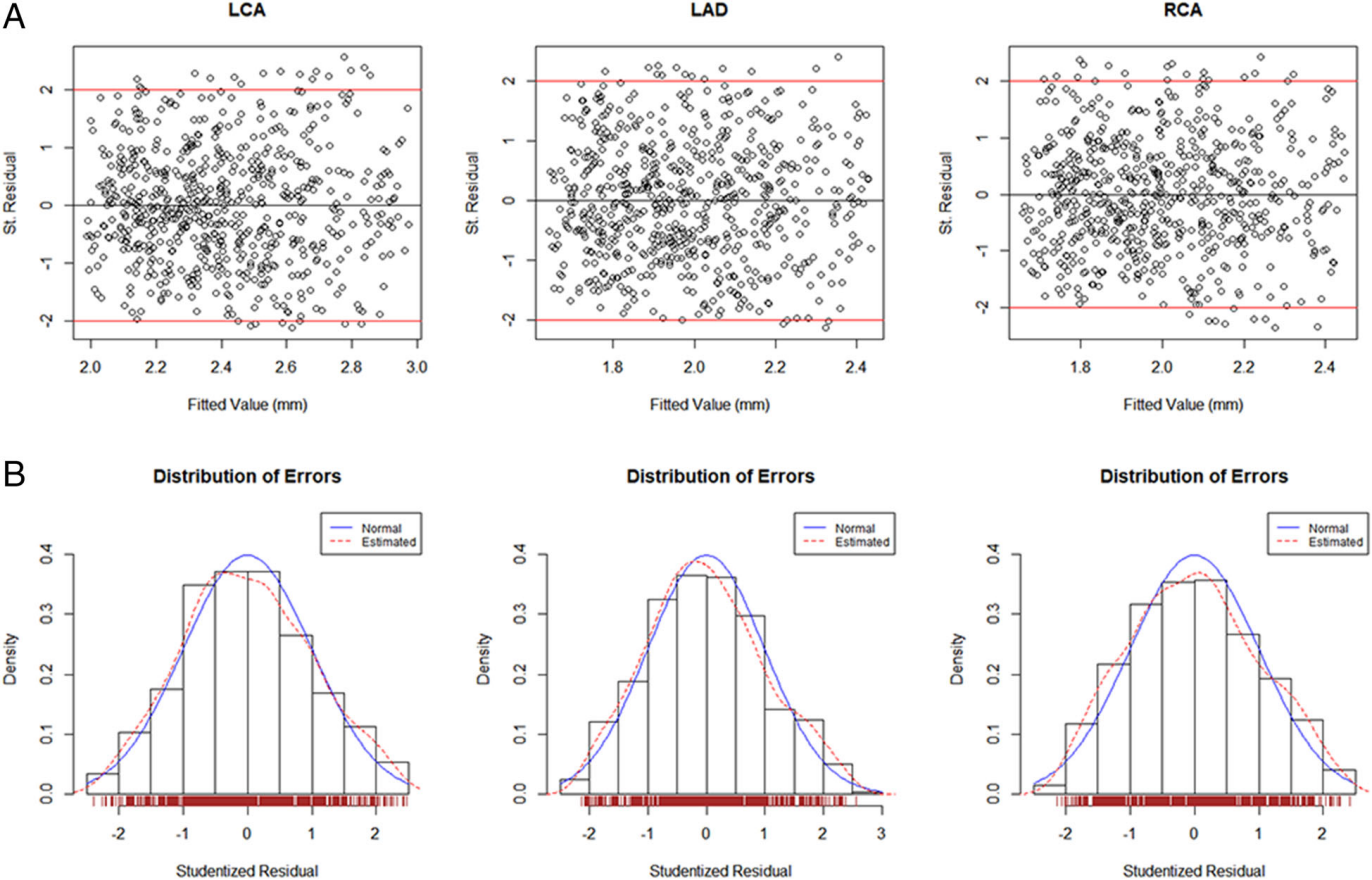
A1 ~ A3: D method *Z* score scatter plots for LCA, LAD, and RCA with 2*Z* score boundaries. B1 ~ B3: studentized residual diagrams for LCA, LAD, and RCA. The black line is the predicted value, and the red line is the prediction interval with 95% confidence

Within certain BSAhay ranges, D method Z scores were non-normally distributed (Fig. 3).

### Performance of the SZ versus D methods

**Fig. 4:**
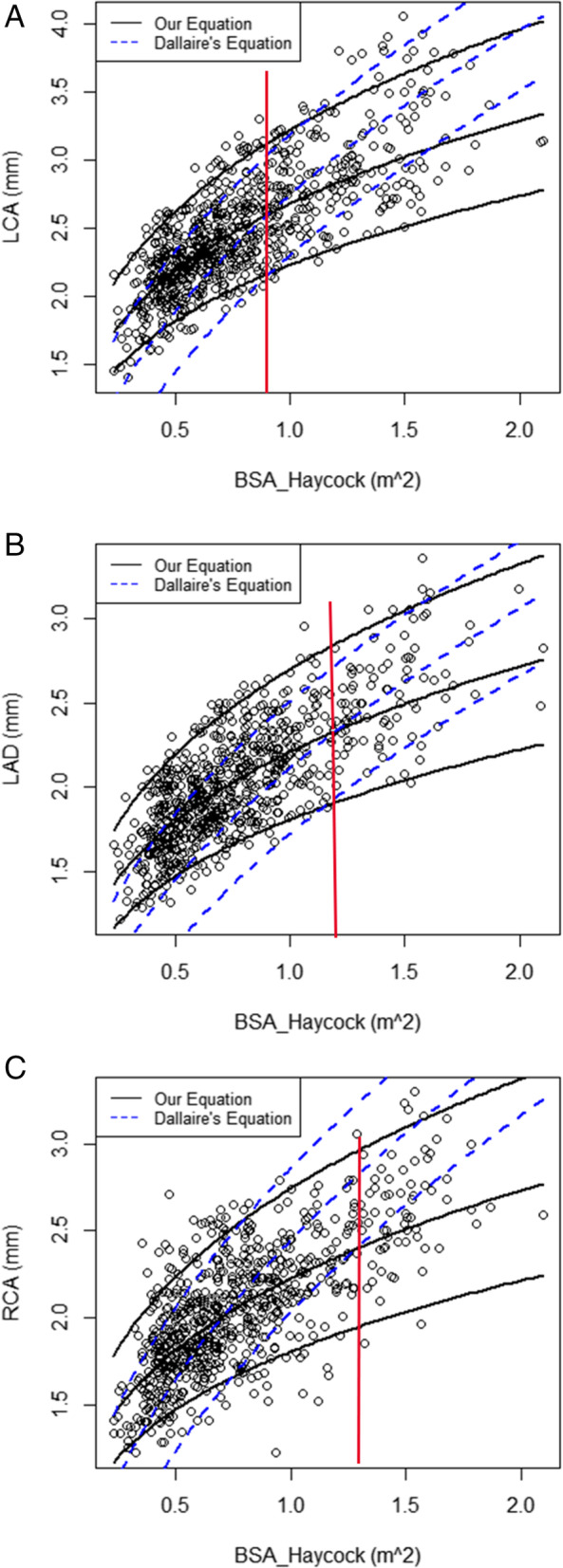
**A**, **B**, **C** Means±2Z scores predicted by the SZ and D methods for LCA, LAD, and RCA. The solid black line represents values predicted by the SZ method, the dotted blue line represents values predicted by the D method, and the solid red line represents the BSA in which the two predicted mean curves intersect. The red vertical line indicates the point where the SZ and D models yield the same z-score prediction for that specific BSA

Within certain BSAhay ranges, the SZ and D methods predicted different values (*P* < 0.05) (Fig. 4). Specifically, D method-predicted LCA diameter means were larger than those predicted by the SZ method when BSAhay> 0.9 m^2^, D method-predicted LAD artery diameter means were smaller than those predicted by the SZ method when BSAhay ≤1.2 m^2^, and D method-predicted means of both diameters were larger than those predicted by the SZ method when BSAhay> 0.6 m^2^.

## Discussion

Because China is large and exhibits regional diversity in individual lifestyle, height, and weight, a multicenter study was conducted to more accurately ascertain pediatric coronary artery diameter reference values. The present study is the largest such multicenter Chinese pediatric study to date, and includes coronary artery measurement data from six regions in Northern and Southern China.

Study findings demonstrate that coronary artery diameters of a healthy Chinese pediatric population increase non-linearly with an increase in BSA, and differ significantly from those of the Dallaire cohort. Specifically, Chinese pediatric coronary artery diameters are larger at lower BSA ranges, but smaller at higher BSA ranges, than those of North American children. In addition, relative to the D method, the SZ method mean reference value-predictive regression model exhibits lower *Z* scores at lower BSA ranges and higher *Z* scores at higher BSA ranges. This suggests that the SZ method may be more accurate than the D method in the Chinese pediatric population.

Previous studies suggested the existence of a linear correlation between equal variance of coronary artery diameter and BSA [14–17]. However, multiple larger studies have found a non-linear correlation between coronary artery diameter and BSA in healthy pediatric populations [2–9, 18]. Coronary artery diameter standard deviation from the mean differs within distinct BSA ranges, resulting in population-inappropriate linear regression methods introducing bias during *Z* score calculation. Therefore, researchers have sought to identify regression models able to more accurately represent the non-linear relationship between coronary artery diameter mean and standard error. Specifically concerning heteroscedastic non-linear relationships, prior studies have evaluated the goodness of fit of various regression models, including quadratic, cubic polynomial, logarithmic, exponential, and square root [2–9].

In the present study, an exponential regression model exhibited the best fit (based on maximum R^2^ and minimum RSE and AIC) and the standard test of normality was therefore applied to model residuals and *Z* scores. Results indicate that the model performs reliably. The present study therefore contributes to establishment of Chinese pediatric coronary artery diameter reference values, and provides data which may help overcome the lack of universality associated with single-center studies.

Up to 25 BSA calculation methods are available, including the Stevenson, Haycock, and Du Bois EF formula [11, 12, 19, 20]. The American Society of Echocardiography guidelines for quantitative pediatric echocardiography recommends use of the Haycock formula when calculating *Z* scores for cardiovascular structure measurements [21, 22]. The Haycock formula may also be a better estimator of BSA for smaller children [4, 22], although different BSA formulae do not result in different model *Z* scores in the present study. However, the Stevenson formula is commonly used within the Chinese healthcare system. Therefore, in order to determine the impact of formula choice on model *Z* scores, the present study derived regression models from a single original dataset (using BSA calculated via either the Stevenson or Haycock formulae), and compared resultant *Z* scores. Because *Z* scores did not differ significantly, either BSA formula is appropriate for use in quantitative evaluation of echocardiography data in a Chinese pediatric population. The present study ultimately incorporated the Haycock formula into the SZ method regression model. This is consistent with the approach of the D method, which also uses the Haycock formula, and facilitates comparison of these two methods.

In comparing *Z* scores resulting from the SZ and D methods, it was found that the SZ method produced *Z* scores closer to zero. This indicates that the SZ method may be more suitable than the D method for use in the Chinese pediatric population. Furthermore, predicted coronary artery diameter mean values and standard deviations differed between North American and Chinese pediatric populations, and the predicted mean value curve provided by the D method had a higher gradient than that provided by the SZ method. When BSA is low, predicted mean values for North American children were lower than those for Chinese children, and when BSA is high, predicted mean values for North American children were higher than those for Chinese children. This indicates that using predictive regression models based on North American pediatric reference values may over- or underestimate coronary artery measurement *Z* scores in the Chinese pediatric population, which would negatively impact CAD diagnosis and treatment.

We suggest that the D method regression model used for the North American pediatric population is unsuitable for use in the Chinese pediatric population. Factors such as region and race should be taken into account when incorporating automatic calculation functions into echocardiographic equipment and when selecting predictive regression models for clinical applications. The SZ method regression model established during the present study may be more suitable for use in the Chinese pediatric population.

However, we acknowledge certain study limitations. For example, determination coefficients (R^2^ values) are marginally lower than those determined by previous studies, especially for the RCA [4, 9]. Determination coefficients for the LCA, LAD artery, and RCA obtained using the D method were higher than those obtained using the SZ method. Two possible reasons may account for this observation: differences between multiple participating study sites, or an unbalanced cohort gender ratio. Furthermore, coronary artery diameters were too small to avoid errors in measurement when enlarging images, and it is challenging to ensure inter-site consistency during a multicenter study. Mitigation strategies for such limitations should be considered when designing future studies.

Furthermore, Study cohort was skewed toward male and there are few subjects older than 12 years of age which limit generalization of this data to children > 12 years of age.

## Conclusions

This study established new coronary artery diameter reference values for echocardiography and a Z score regression equation that hold clinical value for the evaluation of Chinese pediatric populations. A Z score regression equation derived from data of a Caucasian pediatric population may be not suitable for use in chinese population.

## Data Availability

The data that support the findings of this study are available from the authors upon reasonable request.

## Abbreviations

BSA: Body surface area
BSAste: BSA Stevenson
BSAhay: BSA Haycock
CAD: Coronary artery disease
H: Height
W: Weight
SBP / DBP: Systolic / diastolic blood pressure
RCA: Right coronary artery
LCA: Left coronary artery
LAD: Left anterior descending
RSE: Residual standard error
AIC: Akaike information criterion
SZ: ShenZhen
D: Dallaire
